# The effects of volume of interest delineation on MRI-based radiomics analysis: evaluation with two disease groups

**DOI:** 10.1186/s40644-019-0276-7

**Published:** 2019-12-21

**Authors:** Xiao Zhang, Liming Zhong, Bin Zhang, Lu Zhang, Haiyan Du, Lijun Lu, Shuixing Zhang, Wei Yang, Qianjin Feng

**Affiliations:** 1 School of Biomedical Engineering, Southern Medical University, No.1023 Shatai Road, Baiyun District, Guangzhou, 510515 Guangdong China; 2 Department of Radiology, The First Affiliated Hospital, Jinan University, No. 613 Huangpu West Road, Tianhe District, Guangzhou, 510627 Guangdong China; 3 Department of Radiology, Guangdong General Hospital/Guangdong Academy of Medical Sciences, No. 106 Zhongshan Er Road, Yuexiu District, Guangzhou, 510080 Guangdong China

**Keywords:** Radiomics, Magnetic resonance imaging, Breast cancer, Nasopharyngeal carcinoma, Preoperative prediction, Segmentation

## Abstract

**Background:**

Manual delineation of volume of interest (VOI) is widely used in current radiomics analysis, suffering from high variability. The tolerance of delineation differences and possible influence on each step of radiomics analysis are not clear, requiring quantitative assessment. The purpose of our study was to investigate the effects of delineation of VOIs on radiomics analysis for the preoperative prediction of metastasis in nasopharyngeal carcinoma (NPC) and sentinel lymph node (SLN) metastasis in breast cancer.

**Methods:**

This study retrospectively enrolled two datasets (NPC group: 238 cases; SLN group: 146 cases). Three operations, namely, erosion, smoothing, and dilation, were implemented on the VOIs accurately delineated by radiologists to generate diverse VOI variations. Then, we extracted 2068 radiomics features and evaluated the effects of VOI differences on feature values by the intra-class correlation coefficient (ICC). Feature selection was conducted by Maximum Relevance Minimum Redundancy combined with 0.632+ bootstrap algorithms. The prediction performance of radiomics models with random forest classifier were tested on an independent validation cohort by the area under the receive operating characteristic curve (AUC).

**Results:**

The larger the VOIs changed, the fewer features with high ICCs. Under any variation, SLN group showed fewer features with ICC ≥ 0.9 compared with NPC group. Not more than 15% top-predictive features identical to the accurate VOIs were observed across feature selection. The differences of AUCs of models derived from VOIs across smoothing or dilation with 3 pixels were not statistically significant compared with the accurate VOIs (*p* > 0.05) except for T2-weighted fat suppression images (smoothing: 0.845 vs. 0.725, *p* = 0.001; dilation: 0.800 vs. 0.725, *p* = 0.042). Dilation with 5 and 7 pixels contributed to remarkable AUCs in SLN group but the opposite in NPC group. The radiomics models did not perform well when tested by data from other delineations.

**Conclusions:**

Differences in delineation of VOIs affected radiomics analysis, related to specific disease and MRI sequences. Differences from smooth delineation or expansion with 3 pixels width around the tumors or lesions were acceptable. The delineation for radiomics analysis should follow a predefined and unified standard.

## Background

As an emerging non-invasive tool, radiomics has shown gratifying performance in phenotype diagnosis and classification [1, 2], tumor prognosis [3, 4], treatment decision [5, 6], and molecular marker estimation [7, 8] by permitting comprehensive quantification tumor heterogeneity on radiographic imaging [9–11]. The process mainly consists of six consecutive steps including image acquisition, image preprocessing, tumor segmentation, feature extraction, feature selection, and radiomics model development. Each step can be an uncertain factor contributing to an unreasonable result due to a lack of standardization in radiomics analysis. Recent studies have focused on identifying the factors that affect radiomics analysis. Vallieres et al. investigated the impact of six parameters of feature extraction on the prediction of lung metastases in soft-tissue sarcomas of the extremities [12]. Lu et al. evaluated the effects of segmentation and discretization methods on radiomics features in 2-deoxy-2-[18F] fluoro-D-glucose and [11C] methyl-choline positron emission tomography/computed tomography (PET/CT) imaging of nasopharyngeal carcinoma (NPC) [13]. In the process of image preprocessing for patients with head and neck cancers, Bagher-Ebadian et al. evaluated changes in radiomics features from images subject to smoothing, sharpening, and noise relative to baseline datasets [14]. In a recent study, Shiri et al. considered the need of reliable feature values against image reconstruction and assessed the variability of radiomics features extracted from multi-scanner phantom and patient PET/CT images over a wide range of different reconstruction settings [15].

Among the factors that affect radiomics analysis, delineation of tumors or lesions occupy an important position, as the volume of interest (VOI) is directly used to extract quantitative features [9]. The accuracy may affect subsequent radiomics analysis. Usually, VOIs are manually outlined by radiologists with labor intensive as well as time-consuming. The work in [16] showed that the delineation of VOIs for radiotherapy currently was imprecise with high inter-operator variability, even for experienced observers. Most prior studies have focused solely on the effects of inter-observer variability in manual tumor delineation to identify radiomics features with high robustness [17, 18]. In fact, quantification of tumor delineation and tolerance assessment of the differences are likely more important in developing standardized research. Recently, Kocal et al. [19] determined the influence of segmentation with margin shrinkage of 2 mm on CT-based radiomics analysis for distinguishing low and high nuclear grade renal clear cell carcinomas (RcCCs). However, in most cases, delineation tends to overestimate the lesion volume to ensure that the entire lesion is identified [20]. The delineation differences that can be accepted and possible influence on radiomics analysis have not been unexplored, requiring quantitative assessment.

The aim of this work is to investigate the effects of delineation of VOIs on each step of radiomics analysis in detail, including feature extraction, feature selection, and prediction performance of radiomics models. Simultaneously, the tolerance of delineation differences of VOIs was assessed for reference in radiomics analysis.

## Methods

### Patients

Two datasets were collected to investigate the effects of delineation of VOIs on radiomics analysis. The first problem is to distinguish whether metastasis occurs in patients with NPC before radiotherapy. In clinical practice, the majority of NPC patients with metastasis before radiotherapy suffer from poor prognosis [21–23]. Hence, it will be beneficial to improve prognosis if the risk of transfer before treatment can be distinguished accurately and take timely intervention on patients with high risk of metastasis. Our study retrospectively recruited 238 patients with NPC who had been diagnosed by histopathology between August 2009 and January 2013. All patients were divided into two groups in accordance with the metastasis status: (i) metastasizing (TM) group with 126 patients; (ii) non-metastasizing (NM) group with 112 patients.

The second problem is the prediction of sentinel lymph node (SLN) metastasis in patients with breast cancer, as described in [24]. It is of great significance using radiomics analysis to predict SLN metastasis for treatment decision making in breast cancer. A total of 146 consecutive patients with histologically confirmed breast cancer between March 2014 and June 2016 were retrospectively enrolled in this work. The patients consisted of two groups on the basis of SLN metastasis: (i) TM group with 55 patients; (ii) NM group with 91 patients. The inclusion criteria are available in Additional file 1: Note S1.

The patients were divided into a strictly training cohort for radiomics model building and an independent validation cohort (25% in NPC group and 33% in SLN group) for evaluating the final prediction performance. The detailed demographic characteristics and clinical information are summarized in Table 1.

**Table 1:** Demographic characteristics and clinical information of two disease groups

Dataset	Characteristic	Training cohort			Independent validation cohort		
NPC group		NM (*n* = 84)	TM (*n* = 95)	*p*-Value	NM (*n* = 28)	TM (*n* = 31)	*p*-Value
	Sex			0.903			0.398
	Male	63	72		21	26	
	Female	21	23		7	5	
	Mean age (SD)	43.95 (11.72)	45.40 (10.26)	0.394	40.54 (9.74)	45.10 (10.27)	0.121
	Histologic grade			< 0.001*			0.009*
	I	3 (3.57%)	0 (0.0%)		1 (3.57%)	0 (0.0%)	
	II	9 (10.72%)	4 (4.21%)		6 (21.43%)	2 (6.45%)	
	III	47 (55.95%)	19 (20.0%)		14 (50.0%)	8 (25.81%)	
	IV	25 (29.76%)	72 (75.79%)		7 (25.0%)	21 (67.74%)	
	Metastatic sites			–			–
	Lung	–	36 (33.03%)		–	11 (30.56%)	
	Liver	–	30 (27.52%)		–	12 (33.33%)	
	Bone	–	43 (39.45%)		–	13 (36.11%)	
SLN group		NM (*n* = 60)	TM (*n* = 37)	*p*-Value	NM (n = 31)	TM (*n* = 18)	*p*-Value
	Mean age (SD)	46.70 (11.85)	46.59 (11.04)	0.935	47.32 (9.18)	50.33 (10.06)	0.339
	Histologic grade			0.261			0.254
	I	7 (11.7%)	2 (5.4%)		7 (22.6%)	1 (5.6%)	
	II	23 (38.3%)	20 (54.1%)		12 (38.7%)	7 (38.9%)	
	III	30 (50.0%)	15 (40.5%)		12 (38.7%)	10 (55.6%)	
	HER2			0.592			0.061
	Positive	21 (35.0%)	11 (29.7%)		6 (19.4%)	8 (44.4%)	
	Negative	39 (65.0%)	26 (70.3%)		25 (80.6%)	10 (55.6%)	
	Ki67 (SD)	36.30 (24.34)	26.32 (15.61)	0.100	30.68 (25.19)	35.06 (27.26)	0.545
	ADC (SD)	0.86 (0.20)	0.82 (0.16)	0.331	0.84 (0.16)	0.86 (0.19)	0.442

*TM* metastasizing, *NM* non-metastasizing, *HER2* human epidermal growth factor receptor 2, *Ki67* proliferation index, *ADC* apparent diffusion coefficient

* *p* <  0.05 representing statistically significant difference in NM and TM group

### Image acquisition protocol

All analyses were carried out in accordance with the relevant guidelines and regulations, and the requirement to obtain informed consent was waived. This retrospective study was approved by the local institutional review board.

- NPC group: All patients had scanned axial contrast- enhanced T1-weighted (CET1-w) and T2-weighted (T2-w) images acquired from a 1.5-T GE scanner (Signa EXCITE HD, TwinSpeed, GE Healthcare, Milwaukee, WI, USA) and a 1.5-T Philips scanner (Achieva, Philips Healthcare, The Netherlands). The GE MRI acquisition parameters were as follows: CET1-w images (TR/TE: 410/Min Full ms, FOV = 230 × 230 mm^2^, NEX = 2.0, slice thickness = 4 mm, spacing = 1 mm); T2-w images (TR/TE: 5000/85 ms, FOV = 230 × 230 mm^2^, NEX = 2.0, slice thickness = 4 mm, spacing = 1 mm). The Philips MRI acquisition parameters were as follows: CET1-w images (TR/TE: 636/20 ms, FOV = 220× 220 mm^2^, NEX = 4.0, slice thickness = 4.5 mm, spacing = 1 mm); T2-w images (TR/TE: 3700/100 ms, FOV = 220 × 220 mm^2^, NEX = 3.0, slice thickness = 5 mm, spacing = 1 mm).
- SLN group: All patients underwent pretreatment T2-weighted fat suppression (T2-FS) and diffusion-weighted images (DWI) scan. The anatomical MRI data were acquired on a 1.5-T MR scanner (Achieva, Philips Healthcare, Best, The Netherlands) equipped with a 4-channel SENSE breast coil in prone position. Axial DWI with bilateral breast coverage were obtained (TR/TE = 5065/66 ms, FOV = 300 × 300 mm^2^, matrix = 200 × 196, slice thickness = 5 mm, slice gap = 1 mm, b values of 0 and 1000 s/mm^2^) by using single-shot spin-echo echo-planar imaging. T2-FS images of breast were collected (TR/TE = 3400/90 ms, FOV = 320 × 260 mm^2^, matrix = 348 × 299, slice thickness = 3 mm, slice gap = 0.3 mm).

### Image pre-processing

**Fig. 1:**
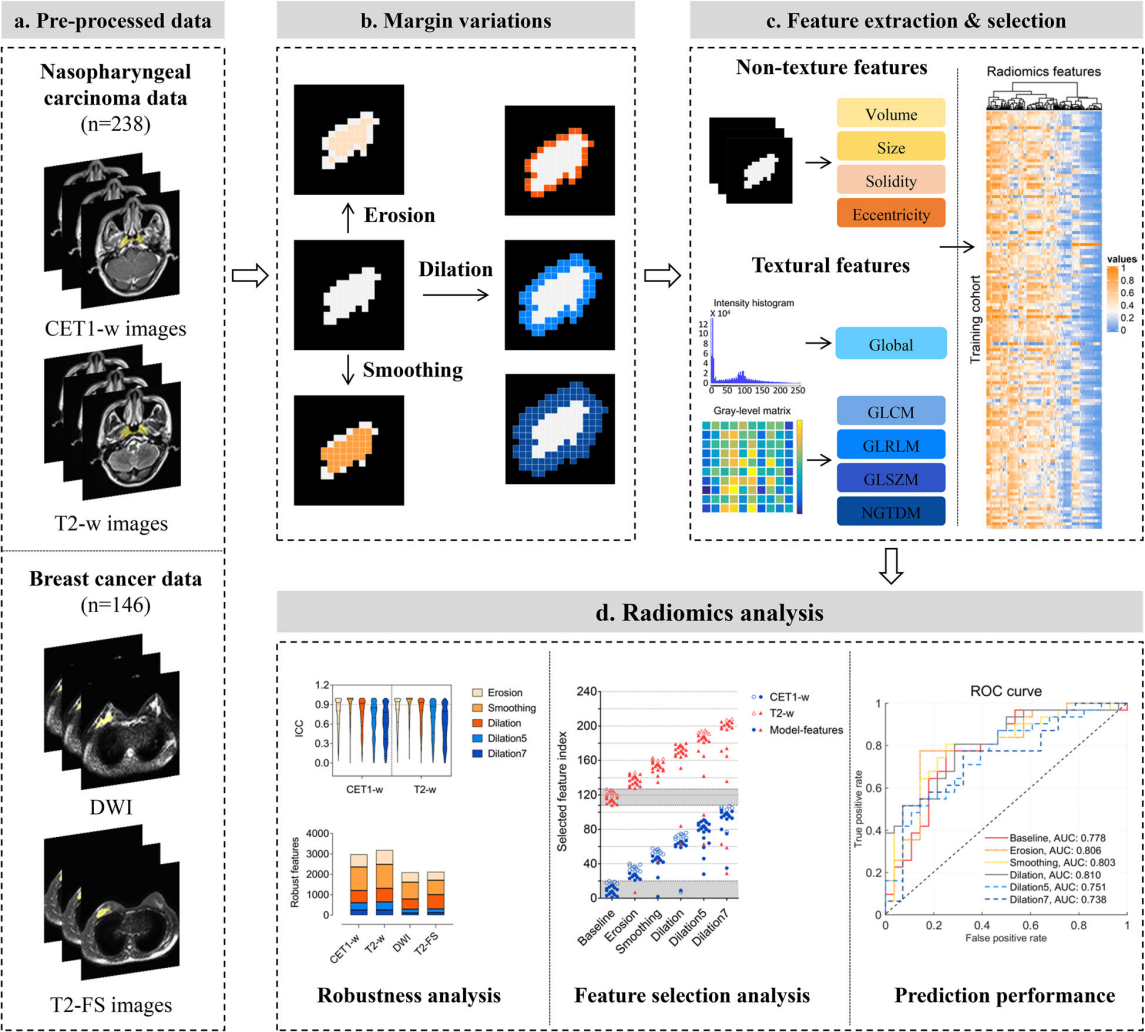
Overall schematic framework of the radiomics analysis: (**a**) MR images across preprocessing of bias field correction and intensity normalization; (**b**) Margin variations consisting of erosion, smoothing, and dilation of various sizes on each slice of volume of interest (VOI), which represent diverse VOI delineations; (**c**) Radiomics features extracted from varying parameter settings and feature selection; (**d**) Radiomics analysis mainly consisting of feature robustness analysis, feature selection analysis, and predictive performance comparison of models from diverse VOIs

As for the subjects in two datasets enrolled in our study, multi-sequence MR images are required from several MR scanners with different protocols, hence image standardization are essential for all images to avoid the inhomogeneity. Prior to analyzing MR images, additional image standardization involving bias field correction and intensity normalization were conducted to avoid inhomogeneity. First, the N4ITK algorithm [25] was applied to remove the bias field artifacts in the MR images. Subsequently, intensity normalization [26] was utilized to reduce the variability across image acquisitions from different manufactures. Fig. 1 illustrates the schematic framework of the radiomics analysis in this work.

### Volume of interest segmentation

All MR images were imported into the ITK-SNAP software designed by Yushkevich et al. [27] to define the VOI of each tumor. The tumor contours were individually first outlined slice-by-slice by two radiologists (Z.L., 4 years of experience, and Z.B., 6 years of experience) and then reviewed by a senior radiologist (Z.S., 12 years of experience). Any disagreement between the readers was discussed until a final consensus was generated. During the session 30 cases randomly selected from each dataset were used for the inter-observer analysis of the segmentation. For each selected region of interest (ROI), the smallest rectangle that best fits the tumor region was used to calculate margin distance of two kinds of manual segmentation in four directions (up, down, left, and right), resulting in multiple calculated values (number of selected ROIs × 4) for analysis together.

### Changes of volume of interest

**Fig. 2:**
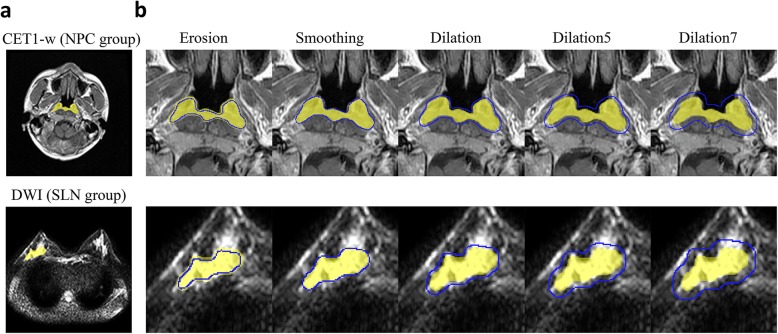
Segmentation of volume of interest and differences in processing: (**a**) Example illustrating the VOI in a single slice of tumor delineated by the radiologists (indicated in yellow); (**b**) Corresponding drawing of partial enlargement under diverse operations (indicated in blue). Erosion represents erosion operation on VOIs while Smoothing represents smoothing operation. Dilation, Dilation5, and Dilation7 indicate dilation operation, for which the size of structural elements is set as 3, 5, and 7, respectively. Application of various operations to VOIs slice-by-slice corresponds to diverse delineations

On the basis of the original segmented regions, erosion, dilation, and smoothing were performed on the VOIs slice-by-slice to generate diverse VOIs. For the dilation operation, the radius sizes (number of pixels) of the circular structural element to dilate the VOIs were separately set as 3, 5, and 7. Given that certain tumors were extremely small, the size for the erosion operation was only set to 3. Image smoothing for VOIs was implemented by a Gaussian smoothing filter configured with correlation operator, where sigma was set as 3 and the template size was 7 × 7. Pixel values outside the bounds of the region of interest were set to the value of the nearest border. The three types of operations were respectively implemented using the functions *imdilate*, *imerode*, and *imfilter* of MATLAB version 8.5 (MathWorks, R2015a). No additional processing was implemented on the contours. For the sake of analysis, five operations on VOIs were abbreviated as Erosion, Smoothing, Dilation, Dilation5, Dilation7, respectively. The VOIs accurately delineated by radiologists were denoted as Baseline. Fig. 2 exemplifies the VOI in a single slice of the original tumor and presents the corresponding drawing of partial enlargement under different operations simultaneously. The degree of tumor volume change of diverse delineations in relation to the accurate delineation is summarized in Table 2.

**Table 2:** Tumor volume change under diverse operations in relation to the accurately outlined tumor

Dataset		Erosion	Smoothing	Dilation	Dilation5	Dilation7
NPC group	CET1-w	0.767 (0.757–0.777)	0.991 (0.989–0.992)	1.350 (1.334–1.366)	1.606 (1.579–1.634)	1.976 (1.932–2.021)
	T2-w	0.745 (0.734–0.757)	0.987 (0.985–0.988)	1.388 (1.369–1.407)	1.675 (1.641–1.708)	2.089 (2.035–2.144)
SLN group	DWI	0.664 (0.639–0.674)	0.959 (0.952–0.964)	1.578 (1.531–1.598)	2.080 (1.979–2.107)	2.858 (2.654–2.882)
	T2-FS	0.788 (0.773–0.802)	0.985 (0.983–0.987)	1.333 (1.307–1.358)	1.609 (1.560–1.658)	2.019 (1.933–2.105)

Note that the value (mean with 95% confidence interval) in the table represents the ratio of the tumor volume after corresponding operations to the original volume

*Erosion* erosion operation, *Smoothing* smoothing operation, *Dilation* dilation with structural element radius size of 3, *Dilation5* dilation with structural element radius size of 5, *Dilation7* dilation with structural element radius size of 7

### Feature extraction

A total of 2068 radiomics features were extracted for each VOI. In reference to [12], four non-texture features that describe the geometric characteristics were calculated, including tumor volume, size, solidity, and eccentricity. In view of the effects of varying extraction parameters on texture features, three extraction parameters, respectively, isotropic voxel size, quantization of gray levels, and quantization algorithm, were adopted, thereby leading to 2064 textural features for each patient. The textural features consisted of Global (extracted from the intensity histogram with 100 bins of the tumor region), grey-level co-occurrence matrix (GLCM), grey-level run length matrix (GLRLM), grey-level size zone matrix (GLSZM) and neighbourhood grey-tone difference matrix (NGTDM) [28–30]. The extraction was conducted with a MATLAB toolkit for radiomics analysis (https://github.com/mvallieres/radiomics). The detailed extraction parameters and description are available in Additional file 1: Note S2 and Table S1.

### Feature selection

The feature selection was performed within the training cohort. Maximum Relevance Minimum Redundancy (mRMR) [31], which has good trade-off between the maximum relevance and minimum redundancy, was firstly explored to identify a well-ranked feature set that included 100 features. Referring to [12, 32], the 0.632+ bootstrap method combined with the area under the receiver operating characteristic curve (AUC) metric were adopted to evaluated the predictability of features (Additional file 1: Note S3). One thousand iterations were performed with 63.2% random data resampling from the training cohort between runs. The features selected in the previous step were ranked through maximizing the 0.632+ bootstrap AUC to determine the final twenty top-predictive features that maximally distinguished two classes.

### Development of radiomics model

Once the discriminative features were identified, radiomics models were built based on different feature sets. A new feature set was composed when one feature from higher to lower rank was added, which contributed to 20 radiomics models. We used the random forest classifier [33] to evaluate the capability of foregoing radiomics models across 10-fold cross-validation in the training cohort, with 150 decision trees used for training ultimately. The model that possessed the most superior properties was determined for further analysis.

### Statistical analysis

First, Mann-Whitney U test was used to compare the difference in age and other continuous variables between TM and NM. Chi-square test was performed to analyze the differences based on factors, such as gender and clinical stages. Statistical analysis was performed on SPSS version 22.0 (IBM, Armonk, NY, USA).

The robustness of features against delineation differences versus the accurate VOIs was quantified using the intra-class correlation coefficient (ICC). Features with ICC ≥ 0.9 were considered excellent robust. The performance of diverse VOI-derived radiomics models were assessed by AUC, and the differences were compared by the method of DeLong et al. [34] using the MedCalc version 15.2.2 (MedCalc Software bvba, Ostend, Belgium). Note that a two-tailed *p* value less than 0.05 indicated statistical significance in this work.

## Results

**Fig. 3:**
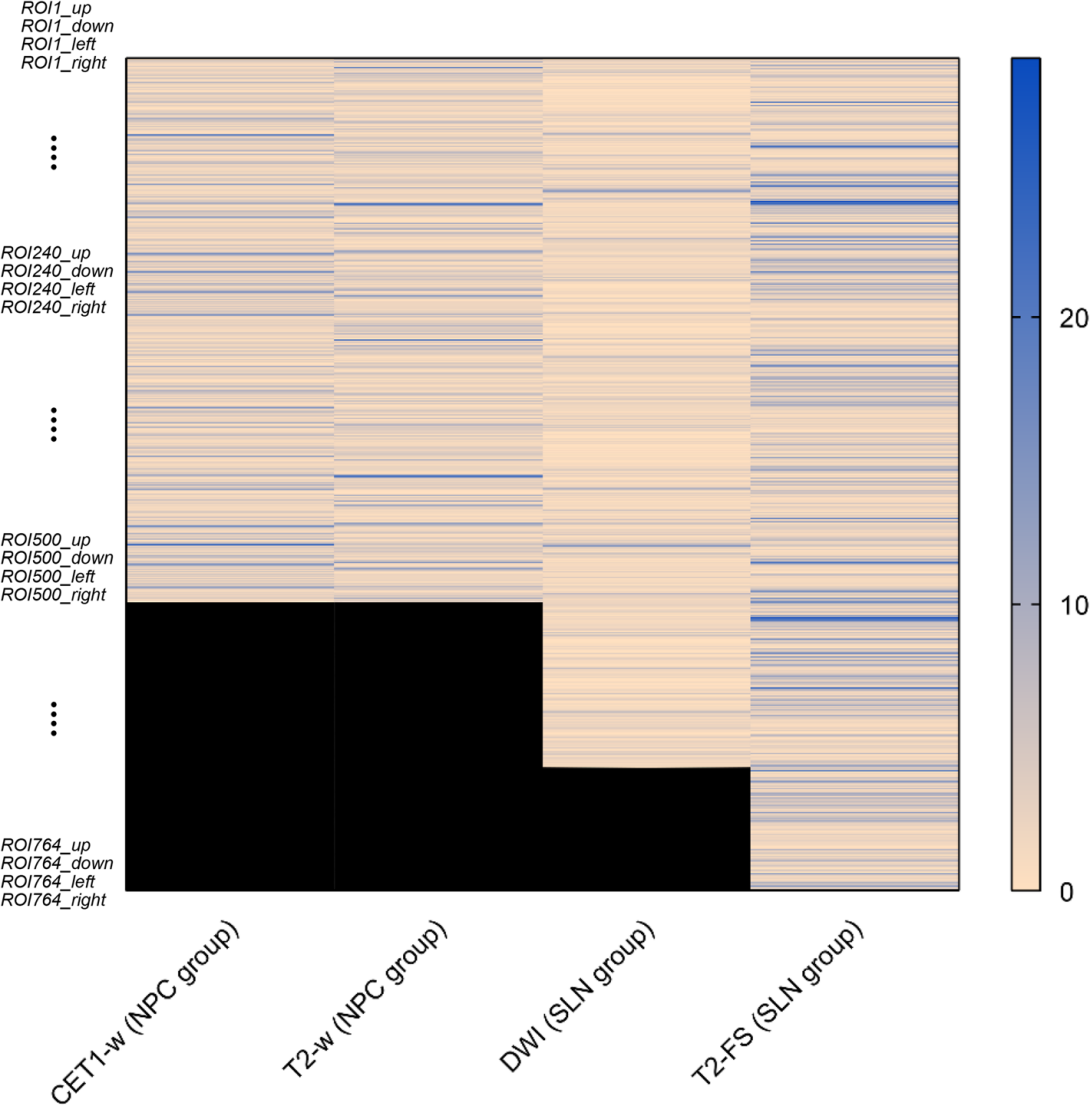
Heatmap for the inter-observer analysis of the segmentation. 30 cases with segmentation by two radiologists were randomly selected from each dataset. For each selected region of interest (ROI), the smallest rectangle that best fits the tumor region was used to calculate margin distance of two kinds of manual segmentation in four directions (up, down, left, and right), resulting in multiple calculated values (number of selected ROIs × 4) for analysis together. Colors in the heatmap indicated that margin differences of ROIs were concentrated between 0 and 8 pixels for all datasets

For the metastasis differentiation in NPC before radiotherapy, no significant differences were observed between NM and TM groups except in histologic grade (*p* <  0.05; Table 1). In the prediction of SLN metastasis in breast cancer, NM and TM groups had no significant differences in all characteristics (*p* > 0.05; Table 1). Inter-observer differences are summarized in Fig. 3. Colors in the heatmap indicated that margin differences of ROIs from two radiologists were concentrated between 0 and 8 pixels for all datasets.

### Feature robustness analysis

**Fig. 4:**
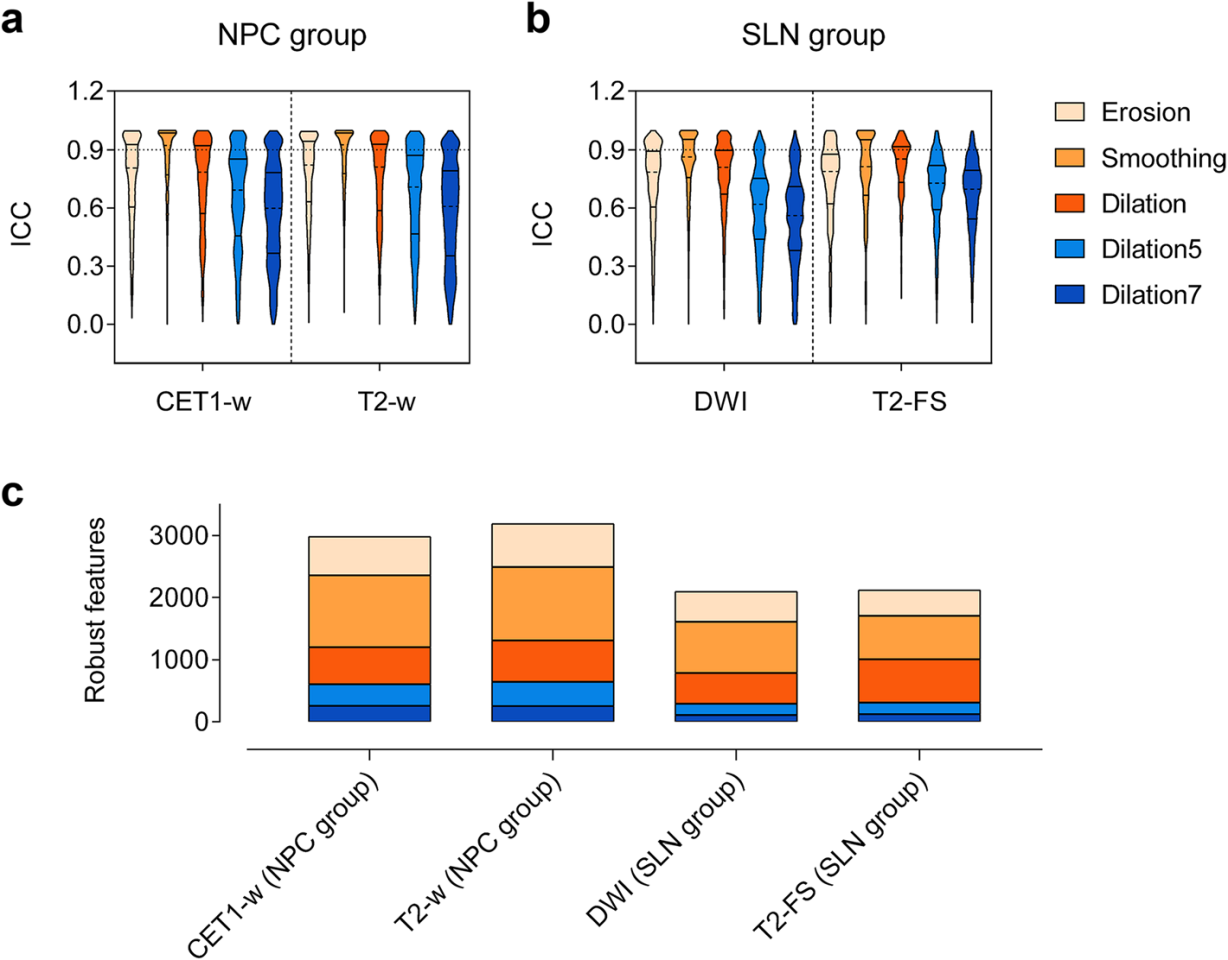
Comparison of the effects of VOI differences on radiomics feature values. ICC violin plots of the radiomics features derived from diverse VOIs for (**a**) NPC group and (**b**) SLN group. The dashed lines represent the median, and the solid lines represent interquartile range. (**c**) Number of robust features identified from diverse VOIs. Features with ICC ≥ 0.9 were considered robust

ICCs for features against all VOI variations were distributed in a wide range for all scans (Fig. 4a and b). ICC values in Smoothing which represented the smallest differences were most concentrated with the smallest effect on feature values, except for T2-FS images, for which Dilation had more concentrated distribution with the narrowest ICC range of 0.134–0.999. Dilation7 which changed the most in VOIs, revealed the largest ICC range with the great effect on feature values. The features extracted from breast cancer data were more sensitive to VOI variations compared with NPC, showing fewer robust features as a whole (Fig. 4c). Smoothing resulted in the maximum number of robust features, whereas Dilation7 worked the other way around.

### Feature selection analysis

**Fig. 5:**
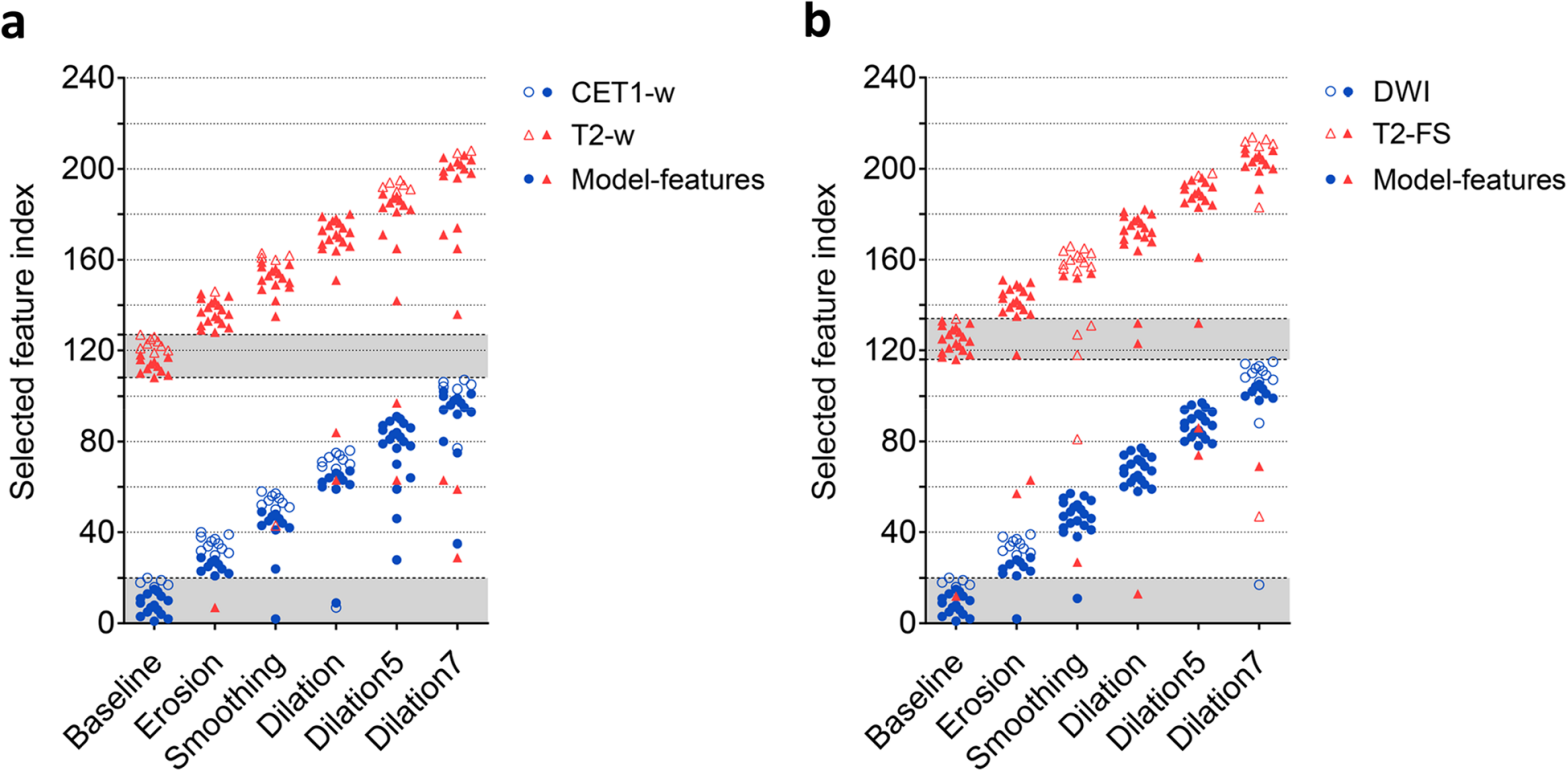
Top-predictive features across feature selection from diverse VOIs: (**a**) NPC group and (**b**) SLN group. Each symbol represents one type of feature, and the area filled with gray represents the range of feature types selected using accurate VOIs. Twenty top-predictive features are identified for each scan, and features in area filled with gray represent the same top-predictive features as accurate VOIs. Note the solid-filled symbols represent features that contribute to the best radiomics models

As a matter of convenience, the top-predictive features selected from diverse VOIs were re-indexed according to feature type. Each symbol in Fig. 5 represents one type of feature, and features in area filled with gray represent the same top-predictive features as accurate VOIs. The features selected under diverse VOIs showed considerable differences (Fig. 5), which indicated great effects of delineation differences on feature selection. Under any variation in the two tasks, not more than 15% top-predictive features were identical to the accurate VOIs, particularly for CET1-w images. This result was the case for no common features. Analogously, there was a large difference in features contributing the best radiomics models (see solid-filled symbols in Fig. 5).

### Prediction performance analysis

As seen in Table 3, the differences of AUCs in Smoothing and Dilation models were not statistically significant with the Baseline model except for T2-FS images in SLN group, the average AUCs of which were much higher (Smoothing: 0.845 vs. 0.725, *p* = 0.001; Dilation: 0.800 vs. 0.725, *p* = 0.042). Erosion, which performed similarly to Smoothing and Dilation model in NPC group, performed the worst in SLN group, especially for DWI with significant differences in comparison with Baseline model (*p* <  0.001). Besides, Dilation5 and Dilation7 model contributed remarkable predictive AUCs in SLN group but the opposite in NPC group. The prediction performance of the training cohorts is shown in Additional file 1: Table S2.

**Table 3:** Prediction results of radiomics models from diverse VOIs on the independent validation cohorts

Dataset	Image	Radiomics model	Feature number	AUC	95% CI	*p*-Value	SEN	SPE	ACCU
NPC group	CET1-w	Baseline	15	0.778	0.720–0.830	–	77.4%	75.0%	72.7%
		Erosion	9	0.806	0.750–0.855	0.403	77.4%	85.7%	76.9%
		Smoothing	11	0.803	0.746–0.852	0.412	80.6%	75.0%	74.1%
		Dilation	10	0.810	0.754–0.858	0.268	80.6%	71.4%	72.0%
		Dilation5	20	0.751	0.691–0.805	0.437	87.1%	53.6%	68.1%
		Dilation7	14	0.738	0.678–0.793	0.220	77.4%	67.9%	68.3%
	T2-w	Baseline	11	0.748	0.688–0.802	–	74.2%	71.4%	70.8%
		Erosion	19	0.710	0.647–0.767	0.359	71.0%	71.4%	68.3%
		Smoothing	15	0.702	0.639–0.759	0.279	74.2%	60.7%	66.1%
		Dilation	20	0.718	0.656–0.774	0.447	77.4%	60.7%	65.8%
		Dilation5	14	0.596	0.530–0.659	0.003*	54.8%	53.6%	53.4%
		Dilation7	18	0.588	0.522–0.651	0.002*	61.3%	64.3%	57.3%
SLN group	DWI	Baseline	15	0.734	0.666–0.794	–	77.8%	67.7%	69.0%
		Erosion	10	0.536	0.500–0.607	< 0.001*	55.6%	58.1%	54.7%
		Smoothing	20	0.711	0.642–0.773	0.617	72.2%	67.7%	63.9%
		Dilation	20	0.737	0.670–0.798	0.934	77.8%	64.5%	66.7%
		Dilation5	20	0.744	0.677–0.803	0.934	66.7%	80.6%	68.2%
		Dilation7	8	0.789	0.725–0.843	0.269	83.3%	74.2%	72.9%
	T2-FS	Baseline	19	0.725	0.657–0.786	–	61.1%	74.2%	66.5%
		Erosion	20	0.696	0.627–0.760	0.319	55.6%	77.4%	61.0%
		Smoothing	4	0.845	0.787–0.893	0.001*	72.2%	74.2%	70.6%
		Dilation	20	0.800	0.737–0.854	0.042*	66.7%	74.2%	70.8%
		Dilation5	18	0.868	0.813–0.912	< 0.001*	77.8%	87.1%	76.5%
		Dilation7	13	0.802	0.739–0.855	0.028*	83.3%	67.7%	72.9%

*Baseline* no processing with the accurate VOIs, *Erosion* erosion operation, *Smoothing* smoothing operation, *Dilation* dilation with structural element radius size of 3, *Dilation5* dilation with structural element radius size of 5, *Dilation7* dilation with structural element radius size of 7, *AUC* area under receiver operating characteristic curve, *CI* confidence interval, S*EN* sensitivity, *SPE* specificity, *ACCU* accuracy, * *p* < 0.05 with DeLong test representing statistically significant difference in VOI-operated model and Baseline model

### Model performance across testing data from diverse VOIs

**Fig. 6:**
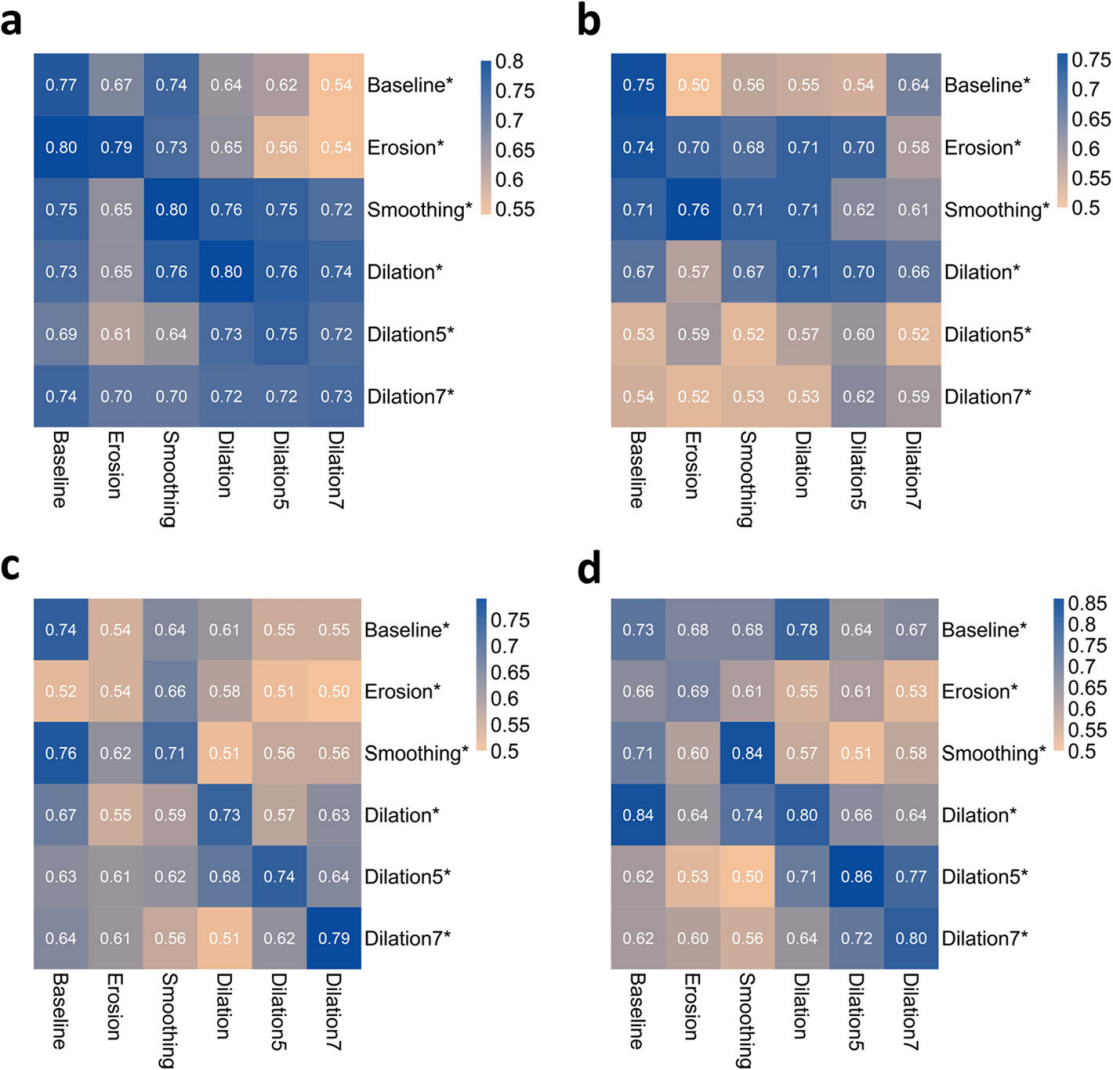
Prediction performance of the radiomics models across different VOIs-operated validation data. NPC group: (**a**) CET1-w images and (**b**) T2-w images; SLN group: (**c**) DWI and (**d**) T2-FS images. Note that the abbreviation marked with an asterisk represents a training model. For example, Erosion* is the training model built with data and parameters obtained from VOIs with erosion. The results in the first row represent the prediction performance of the Baseline model tested by different VOIs-operated data

On the basis of feature parameters obtained from the radiomics models, we assessed stability by validating the model using data from diverse VOIs, as shown in Fig. 6. The predictive AUCs using CET1-w images, trained by data from the Dilation7 model and tested by data from other VOIs, were all above 0.7 and seemed relatively stable. Poor prediction results were still represented by Dilation5 and Dilation7 models using T2-w images. The prediction results changed in relatively large ranges across different VOI-operated validation data in SLN group. The model with training and validation data undergoing the same delineation outperformed other models in most cases.

## Discussion

In this work, we investigated the influence of tumor delineation on radiomics analysis in detail within two disease groups. The tolerance of the delineation differences was explored to provide references for tumor delineation in future radiomics studies. Application of various operations to VOIs corresponded to diverse delineations in clinical practice. The results illustrated that delineation differences of VOIs had an effect on the radiomics feature values, feature selection, and prediction performance which depended on specific disease as well as MRI sequences.

The experiment results provided strong evidence that the larger the VOIs changed, the greater the influence on the feature values (Fig. 4). According to the number of robust features, different diseases had discrepant sensitivity to VOI variations, consistent with a previous discovery [18]. This result could be explained from the fact that the tumors in breast cancer are larger with ill-defined margins, which cause great changes on the feature values across larger variation. A comparison of top-predictive features showed that even slight smoothing on VOIs could lead to large differences in feature selection. This agrees with the discovery in [19], only one texture feature appeared on both contour-focused segmentation and the one with shrinkage of 2 mm. Probably because the variations exactly weaken the correlation with the class of certain features by changing the feature values, which resulted in a new order of top-predictive features. A feature possessing good distinguishing characteristics does not stand out under all conditions, and thus depended on the specific analysis task.

The study also demonstrated that delineation differences of VOIs affected prediction performance of radiomics models. Stable and prominent performance from VOIs across Smoothing and Dilation indicated the tolerance of corresponding differences for radiomics models and corroborated the feasibility that the radiologists smoothly outline the lesions or slightly larger of 3 pixels width around the tumor. Note that it is not that bigger is better for VOIs. The worse performance from VOIs across Dilation5 and Dilation 7 in NPC group (Fig. 2) could be explained by the dilated area that contained more areas of the nasal cavity which exhibits low-signal intensity. This increased the effect of certain features, tending to confusion classification and facilitating feature sets with poor differentiation property. However, in breast images, more soft tissues containing complex textures were associated to capture heterogeneity for predicting SLN metastasis, indicating that the peritumoral regions had a positive influence to a certain extent. This finding is consistent with past researches [35, 36]. Braman et al. showed that the textural analysis of peritumoral regions contributed to the prediction of pathological complete response in neoadjuvant chemotherapy on pretreating breast cancer DCE-MRI [36]. This explanation also holds true for the worse performance from VOIs across erosion.

The radiomics models with good predictive properties might not necessarily perform well on the validation data from VOIs of diverse delineations, which implied that the VOIs of training and validation data should be outlined on the basis of the same criterion. In this regards, a unified standard should be referred in the delineation of VOIs, e.g., slight larger delineation with 3 pixels width around the tumors or lesions for all images. We suppose this assists in more accurate analysis, as the same proposal by Welch et al. [37]. In particular, the Dilation7 model distinctly reflected stable performance against all the variations using CET1-w images. The modeling features are shown in Additional file 1: Table S3. Beyond our expectation, no features showed high robustness, whether in one or all variations. We can infer that features which are not robust to the differences in VOIs may not result in poor prediction performance, which is similar with the observation of past researches [38, 39]. The results also confirmed the insufficiency of simply analyzing the effects of differences on features robustness. In fact, whether the final performance of the radiomics models exist substantial differences is the most important issue, as emphasized in [40].

The present work also has several generalizability issues and limitations. First, while the number of patient population was small, an independent validation cohort was divided for radiomics model evaluation devoid of information leakage between feature selection/training phases. We believe this makes the results reliable and generalizable. It is in demand of more patient data for stronger verification in future research. Second, we used simple morphological operations to change tumor margin. Other contour randomization processing methods that provide stochastic components in the delineation of VOIs are lacking. For the purpose of determining the feasibility of alternative delineation of VOIs, relative changing of the tumor-focused delineation is easier to implement from a medical point of view. Third, regarding the differences in image resolutions between MR images, we changed the size of VOIs at pixel level to better adapt to the delineation of different scenarios. As radiologists delineate the VOIs in term of the original images, which does not involve image resampling and additional preprocessing. Fourth, the effects of the diverse delineations were analyzed and synthesized in spite of differences in tumor location and imaging manifestations within two disease groups. More types of diseases should be further assessed to provide more comprehensive references. Additionally, we only assessed the effects of diverse delineations of VOIs using MRI. The effect on the radiomics analysis for other modalities, such as PET/CT, is still unclear.

## Conclusions

The differences in delineation of VOIs could lead to considerable differences in feature value and feature selection. The influence on prediction depended on specific disease as well as MRI sequences, among which smooth or slight larger delineation with 3 pixels width around the tumors or lesions were feasible. In addition, predefining a unified standard is suggested in the delineation to promote reliable analysis. Despite several limitations, we believe these findings are of great significance as a reference for tumor delineation in future radiomics analysis.

## Supplementary information

**Additional file 1. Note S1** : the inclusion criteria and flow chart of the patient data. **Note S2**: radiomics features. **Note S3**: 0.632+ bootstrap feature selection. **Figure S1.** The inclusion criteria and flow chart of nasopharyngeal carcinoma data. **Figure S2.** The inclusion criteria and flow chart of breast cancer data. **Table S1.** Radiomics features type and number. **Table S2.** Prediction results of radiomics models from diverse VOIs on the training cohorts of two disease groups. **Table S3.** Radiomics features for the Dilation7 (dilation with structural element radius size of 7) model as well as the ICCs for metastasis estimation in nasopharyngeal carcinoma

## Data Availability

The data that support the findings of this study are available from corresponding author upon reasonable request.

## Abbreviations

AUC: Area under the ROC curve
CET1-w: Contrast-enhanced T1-weighted
CT: Computed tomography
DWI: Diffusion-weighted image
GLCM: Grey-level co-occurrence matrix
GLRLM: Grey-level run length matrix
GLSZM: Grey-level size zone matrix
ICC: Intra-class correlation coefficient
MRI: Magnetic resonance imaging
NGTDM: Neighbourhood grey-tone difference matrix
NM: Non-metastasizing
NPC: Nasopharyngeal carcinoma
PET: Positron emission tomography
ROC: Receiver operating characteristic
ROI: Region of interest
SLN: Sentinel lymph node
T2-FS: T2-weighted fat suppression
T2-w: T2-weighted
TM: Metastasizing
VOI: Volume of interest
