# Supplementary material for: The effects of volume of interest delineation on MRI-based radiomics analysis: evaluation with two disease groups
Source: Cancer Imaging. 2019 Dec 21;19:89. doi: 10.1186/s40644-019-0276-7 (PMC6925418; doi:10.1186/s40644-019-0276-7)
Supplement: Supplementary file 1 — Additional file 1. Note S1 : the inclusion criteria and flow chart of the patient data. Note S2: radiomics features. Note S3: 0.632+ bootstrap feature selection. Figure S1. The inclusion criteria and flow chart of nasopharyngeal carcinoma data. Figure S2. The inclusion criteria and flow chart of breast cancer data. Table S1. Radiomics features type and number. Table S2. Prediction results of radiomics models from diverse VOIs on the training cohorts of two disease groups. Table S3. Radiomics features for the Dilation7 (dilation with structural element radius size of 7) model as well as the ICCs for metastasis estimation in nasopharyngeal carcinoma [file 40644_2019_276_MOESM1_ESM.docx]

**Supplementary Material**

## Supplementary note 1: the inclusion criteria and flow chart of the patient data

- **Nasopharyngeal carcinoma data**

**
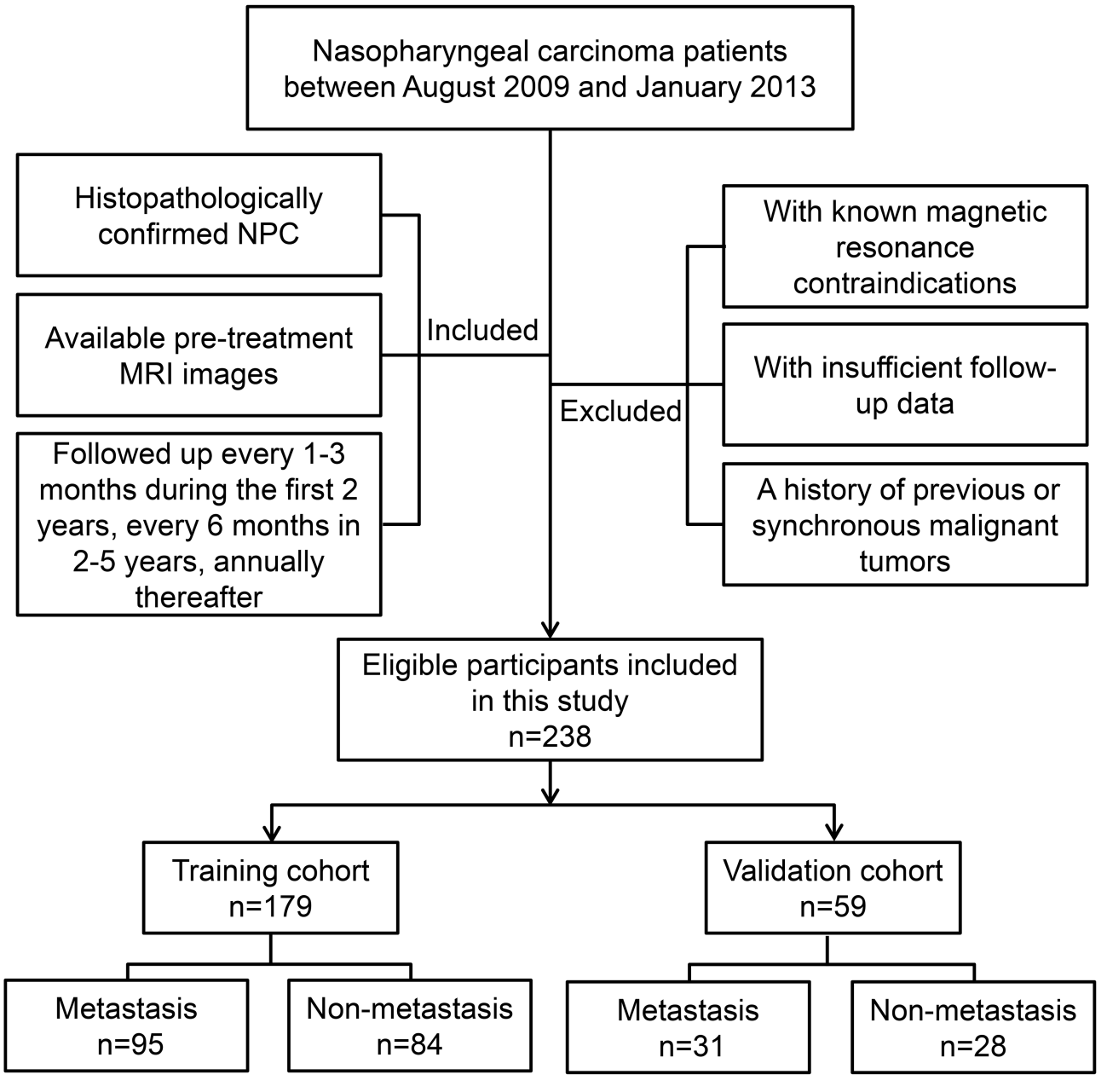
**

**Fig S1** The inclusion criteria and flow chart of nasopharyngeal carcinoma data

- **Breast cancer data**

**
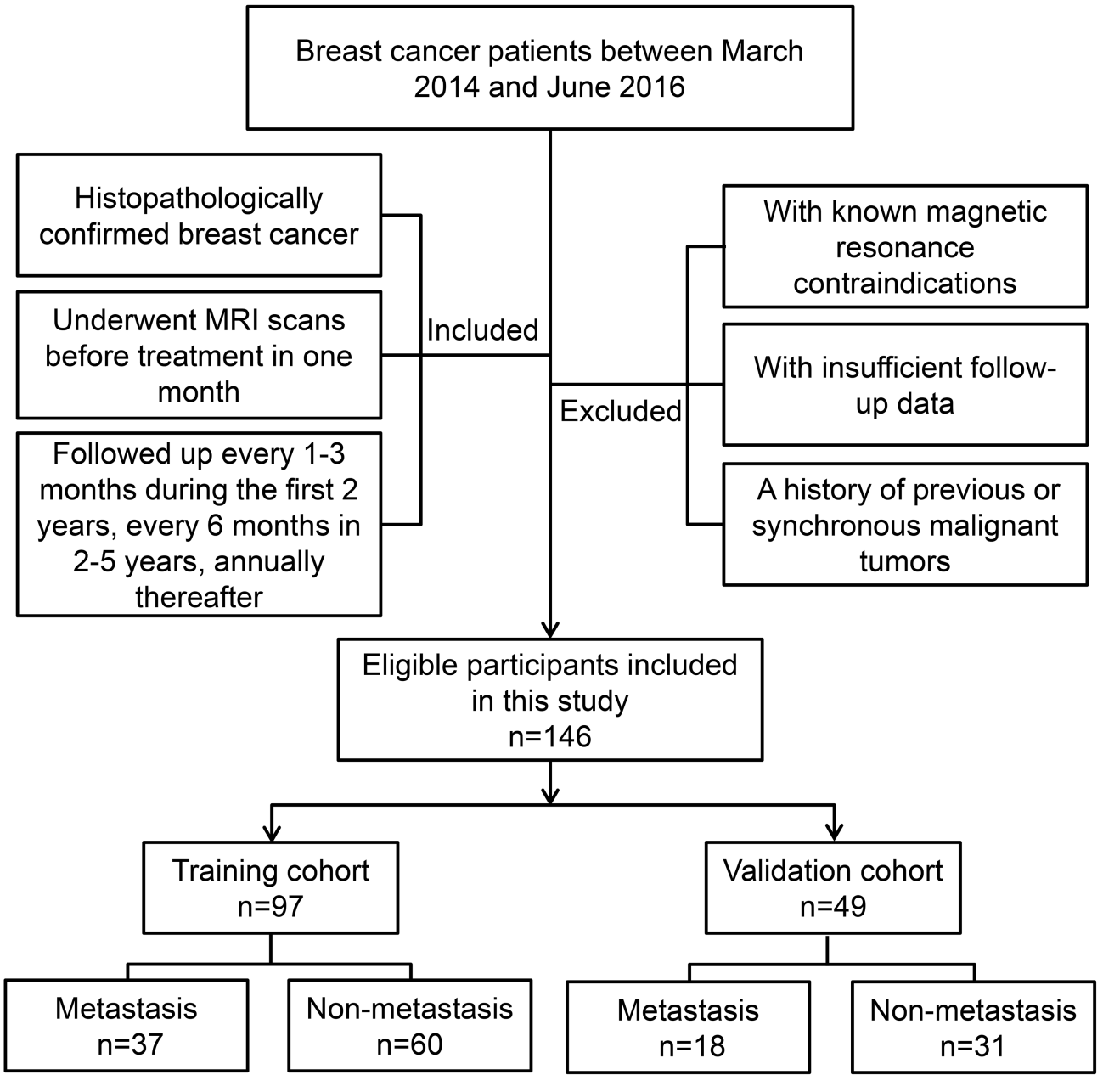
**

**Fig S2** The inclusion criteria and flow chart of breast cancer data

# Supplementary note 2: radiomics features

In feature extraction, our study allowed for extraction of texture features at varying image standardization parameter settings. The overwhelming majority of radiomics analysis extracted texture features at fixed parameters, which may not be optimal even if one-sided. Still, voxel size, intensity quantization method, and gray level significantly influenced texture features [1, 2]. However, no parameter setting was optimal for all features with good property. As such, extracting features at individually optimal parameter settings is better than fixed parameters. We extracted 43 texture features at different parameter settings, which generated 2048 features for building the radiomics model. This method could potentially improve the prediction performance of radiomics analysis.

Global features were extracted from the intensity histogram with 100 bins of the tumor region, whereas GLCM, GLRLM, GLSZM and NGTDM textures were matrix-based features. Considering the effects of different parameters on texture feature values, three extraction parameters explored in our study included the following:

1) *Isotropic voxel size*, the values of which investigated in our study is 1 mm, 2 mm, 3 mm, 4 mm, 5 mm and initial in-plane resolution (denoted as pixelW), thus voxels in each volume were resampled to an isotropic voxel size set to a desired resolution using cubic interpolation;

2) *Quantization of gray levels*, converting the full intensity range of the region of interest to a smaller number of gray levels, in which 8, 16, 32 and 64 for number of gray levels were tested;

3) *Quantization algorithms*, including Equal-probability and Lloyd-Max quantization algorithms implemented in this work.

Thus, the texture features were extracted at different combinations of the image standardization parameters, leading to a total of 2068 radiomics features for each patient, see Table S2.

The specific calculation formulas of features were as follows:

- **Non-texture features:**

1. Volume: Number of voxels in the tumor region multiplied by the dimension of voxels.

2. Size: Longest diameter of the tumor region.

3. Solidity: Ratio of the number of voxels in the tumor region to the number of voxels in the 3D convex hull of the tumor region.

4. Eccentricity: ${[1-a\times b/c^{2}]}^{1/2}$, where *c* is the longest semi-principal axes of the ellipsoid that best fits the tumor region, and *a*, *b* are separately the second and third longest semi-principal axes of the ellipsoid.

- **Global:**

Global features are extracted from the intensity histogram of ROI. Here, histograms with 100 bins were used for the computation of global features in this work. Let: $p_{i}$ be the gray level probability with gray level$i$, $x_{i}$ be the number of voxels, $\mu$ be the average value, $\sigma$ be the standard deviation.

1. Variance

$$\mathrm{var}iance=\sum_{i=1}^{n} p_{i}\left(x_{i}-\mu\right)^{2}$$

1. Skewness

$$Skewness=\frac{\sum\left(x_{i}-\mu\right)^{3}}{n\sigma^{3}}$$

1. Kurtosis

$$Kurtosis=\frac{\sum\left(x_{i}-\mu\right)^{4}}{n\sigma^{4}}$$

- **Gray-level co-occurrence matrix (GLCM):**

GLCMs are calculated per scan in 13 directions of 3D spaces as referred to [3]. Let：$P(i,j)$ be the co-occurrence matrix for an arbitrary $\delta$and $\theta$, $p(i,j)$ be the normalized co-occurrence matrix, $N_{g}$ be the number of discrete intensity levels in the image, $p_{x}(i)$ be the marginal row probabilities, $p_{y}(j)$ be the marginal column probabilities, $\mu_{x}$ be the mean gray level intensity of $p_{x}$, $\mu_{y}$ be the mean gray level intensity of $p_{y}$, $\sigma_{x}$ be the standard deviation of $p_{x}$, $\sigma_{y}$ be the standard deviation of $p_{y}$.

$$p(i,j)=\frac{P(i,j)}{\sum P(i,j)},\text{ }p_{x}(i)=\sum_{j=1}^{N_{g}} P(i,j),\text{ }p_{y}(j)=\sum_{i=1}^{N_{g}} P(i,j)$$

$$\mu_{x}=\sum_{i=1}^{N_{g}} p_{x}(i)i,\text{ }\mu_{y}=\sum_{j=1}^{N_{g}} p_{y}(j)j$$

$$\begin{matrix} p_{x+y}(k)=\sum_{i=1}^{N_{g}} \sum_{j=1}^{N_{g}} p(i,j),\text{ }where\text{ }i+j=k,\text{ }and\text{ }k=2,3,\ldots,2N_{g} \\ p_{x-y}(k)=\sum_{i=1}^{N_{g}} \sum_{j=1}^{N_{g}} p(i,j),\text{ }where\text{ }\left|i-j\right|=k,\text{ }and\text{ }k=0,1,\ldots,N_{g}-1 \end{matrix}$$

$$\begin{matrix} HX=-\sum_{i=1}^{N_{g}} p_{x}(i)\log_{2}(p_{x}(i)),\text{ }HY=-\sum_{j=1}^{N_{g}} p_{y}(j)\log_{2}(p_{y}(i)),\text{ }HXY=-\sum_{i=1}^{N_{g}} \sum_{j=1}^{N_{g}} p(i,j)\log_{2}(p(i,j)) \\ HXY1=-\sum_{i=1}^{N_{g}} \sum_{j=1}^{N_{g}} p(i,j)\log_{2}(p_{x}(i)p_{y}(j)),\text{ }HXY2=-\sum_{i=1}^{N_{g}} \sum_{j=1}^{N_{g}} p_{x}(i)p_{y}(j)\log_{2}(p_{x}(i)p_{y}(j)) \end{matrix}$$

1. Energy

$$energy=\sum_{i=1}^{N_{g}} \sum_{j=1}^{N_{g}} [p\left(i,j\right)]^{2}$$

1. Contrast

$$contrast=\sum_{i=1}^{N_{g}} \sum_{j=1}^{N_{g}} \left(i-j\right)^{2}p\left(i,j\right)$$

1. Entropy

$$entropy=-\sum_{i=1}^{N_{g}} \sum_{j=1}^{N_{g}} p\left(i-j\right)\log_{2}[p\left(i,j\right)]$$

1. Homogeneity

$$\hom ogeneity=\sum_{i=1}^{N_{g}} \sum_{j=1}^{N_{g}} \frac{p\left(i,j\right)}{1+\left|i-j\right|}$$

1. Correlation.

$$correlation=\frac{\sum_{i=1}^{N_{g}} \sum_{j=1}^{N_{g}} p\left(i,j\right)ij-\mu_{x}\mu_{y}}{\sigma_{x}(i)\sigma_{y}(j)}$$

1. Sum Average.

$$sum\text{ }average=\frac{1}{N_{g}\times N_{g}}\sum_{i=1}^{N_{g}} \sum_{j=1}^{N_{g}} \left[p\left(i,j\right)i+p\left(i,j\right)j\right]$$

1. Variance

$$\mathrm{var}iance=\frac{1}{N_{g}\times N_{g}}\sum_{i=1}^{N_{g}} \sum_{j=1}^{N_{g}} \left[\left(i-\mu_{x}\right)^{2}p\left(i,j\right)+\left(j-\mu_{y}\right)^{2}p\left(i,j\right)\right]$$

1. Dissimilarity

$$dissimilarity=\sum_{i=1}^{N_{g}} \sum_{j=1}^{N_{g}} \left|i-j\right|p(i,j)$$

1. Auto Correlation

$$auto\text{ }correlation=\sum_{i=1}^{N_{g}} \sum_{j=1}^{N_{g}} p\left(i,j\right)ij$$

- **Gray-level run-length matrix (GLRLM):**

As described in [4-6], GLRLMs quantify gray level runs which are defined as the length in number of pixels, of consecutive pixels that have the same value. GLRLMs were calculated in 3D for 13 directions. Let: $P(i,j|\theta)$ be the run length matrix for an arbitrary direction $\theta$, $p(i,j|\theta)$ be the normalized run length matrix, $N_{g}$ be the number of discreet intensity values in the image, $N_{\tau}$ be the number of discreet run lengths in the image, $N_{p}$ be the number of voxels in the image, $N_{z}(\theta)$ be the number of runs in the image along angle $\theta$.

$$N_{z}\left(\theta\right)=\sum_{i=1}^{N_{g}} \sum_{j=1}^{N_{\tau}} P\left(i,j\left|\theta\right.\right),\text{ }where\text{ }1\leq N_{z}\left(\theta\right)\leq N_{P}$$

$$p\left(i,j\left|\theta\right.\right)=\frac{P\left(i,j\left|\theta\right.\right)}{N_{z}\left(\theta\right)}$$

1. Short Run Emphasis (SRE)

$$SRE=\frac{\sum_{i=1}^{N_{g}} \sum_{j=1}^{N_{\tau}} \frac{P\left(i,j\left|\theta\right.\right)}{j^{2}}}{N_{z}\left(\theta\right)}$$

1. Long Run Emphasis (LRE)

$$LRE=\frac{\sum_{i=1}^{N_{g}} \sum_{j=1}^{N_{\tau}} P\left(i,j\left|\theta\right.\right)j^{2}}{N_{z}\left(\theta\right)}$$

1. Gray-Level Non-uniformity (GLN)

$$GLN=\frac{\sum_{i=1}^{N_{g}} \left(\sum_{j=1}^{N_{\tau}} P\left(i,j\left|\theta\right.\right)\right)^{2}}{N_{z}\left(\theta\right)}$$

1. Run-Length Non-uniformity (RLN).

$$RLN=\frac{\sum_{j=1}^{N_{\tau}} \left(\sum_{i=1}^{N_{g}} P\left(i,j\left|\theta\right.\right)\right)^{2}}{N_{z}\left(\theta\right)}$$

1. Run Percentage (RP)

$$RP=\frac{N_{z}\left(\theta\right)}{N_{p}}$$

1. Low Gray-Level Run Emphasis (LGRE)

$$LGRE=\frac{\sum_{i=1}^{N_{g}} \sum_{j=1}^{N_{\tau}} \frac{P\left(i,j\left|\theta\right.\right)}{i^{2}}}{N_{z}\left(\theta\right)}$$

1. High Gray-Level Run Emphasis (HGRE)

$$HGRE=\frac{\sum_{i=1}^{N_{g}} \sum_{j=1}^{N_{\tau}} P\left(i,j\left|\theta\right.\right)i^{2}}{N_{z}\left(\theta\right)}$$

1. Short Run Low Gray-Level Emphasis (SRLGE)

$$SRLGE=\frac{\sum_{i=1}^{N_{g}} \sum_{j=1}^{N_{\tau}} \frac{P\left(i,j\left|\theta\right.\right)}{i^{2}j^{2}}}{N_{z}\left(\theta\right)}$$

1. Short Run High Gray-Level Emphasis (SRHGE)

$$SRHGE=\frac{\sum_{i=1}^{N_{g}} \sum_{j=1}^{N_{\tau}} \frac{P\left(i,j\left|\theta\right.\right)i^{2}}{j^{2}}}{N_{z}\left(\theta\right)}$$

1. Long Run Low Gray-Level Emphasis (LRLGE)

$$LRLGE=\frac{\sum_{i=1}^{N_{g}} \sum_{j=1}^{N_{\tau}} \frac{P\left(i,j\left|\theta\right.\right)j^{2}}{i^{2}}}{N_{z}\left(\theta\right)}$$

1. Long Run High Gray-Level Emphasis (LRHGE)

$$LRHGE=\frac{\sum_{i=1}^{N_{g}} \sum_{j=1}^{N_{\tau}} P\left(i,j\left|\theta\right.\right)i^{2}j^{2}}{N_{z}\left(\theta\right)}$$

1. Gray-Level Variance (GLV)

$$GLV=\sum_{i=1}^{N_{g}} \sum_{j=1}^{N_{\tau}} p\left(i,j\left|\theta\right.\right)\left(i-\sum_{i=1}^{N_{g}} \sum_{j=1}^{N_{\tau}} p\left(i,j\left|\theta\right.\right)i\right)^{2}$$

1. Run-Length Variance (RLV)

$$RLV=\sum_{i=1}^{N_{g}} \sum_{j=1}^{N_{\tau}} p\left(i,j\left|\theta\right.\right)\left(j-\sum_{i=1}^{N_{g}} \sum_{j=1}^{N_{\tau}} p\left(i,j\left|\theta\right.\right)j\right)^{2}$$

- **Gray-level size zone matrix (GLSZM):**

GLSZMs quantify gray level zones in an image, of which a gray level zone is defined as the number of connected voxels that share the same gray level intensity [4-7]. Let: $P(i,j)$ be the size zone matrix, $p(i,j)$ be the normalized size zone matrix, $N_{g}$ be the number of discreet intensity values in the image, $N_{s}$ be the number of discreet zone sizes in the image, $N_{p}$ be the number of voxels in the image, $N_{z}$ be the number of zones in the ROI.

$$N_{z}=\sum_{i=1}^{N_{g}} \sum_{j=1}^{N_{s}} P\left(i,j\right),where1\leq N_{z}\leq N_{p}$$

$$\text{ }p\left(i,j\right)=\frac{P\left(i,j\right)}{N_{z}}$$

1. Small Zone Emphasis (SZE)

$$SZE=\frac{\sum_{i=1}^{N_{g}} \sum_{j=1}^{N_{s}} \frac{P\left(i,j\right)}{j^{2}}}{N_{z}}$$

1. Large Zone Emphasis (LZE)

$$LZE=\frac{\sum_{i=1}^{N_{g}} \sum_{j=1}^{N_{s}} P\left(i,j\right)j^{2}}{N_{z}}$$

1. Gray-Level Non-uniformity (GLN)

$$GLN=\frac{\sum_{i=1}^{N_{g}} \left(\sum_{j=1}^{N_{s}} P\left(i,j\right)\right)^{2}}{N_{z}}$$

1. Zone-Size Non-uniformity (ZSN)

$$ZSN=\frac{\sum_{j=1}^{N_{s}} \left(\sum_{i=1}^{N_{g}} P\left(i,j\right)\right)^{2}}{N_{z}}$$

1. Zone Percentage (ZP)

$$ZP=\frac{N_{z}}{N_{p}}$$

1. Low Gray-Level Zone Emphasis (LGZE)

$$LGZE=\frac{\sum_{i=1}^{N_{g}} \sum_{j=1}^{N_{s}} \frac{P\left(i,j\right)}{i^{2}}}{N_{z}}$$

1. High Gray-Level Zone Emphasis (HGZE)

$$HGZE=\frac{\sum_{i=1}^{N_{g}} \sum_{j=1}^{N_{s}} P\left(i,j\right)i^{2}}{N_{z}}$$

1. Small Zone Low Gray-Level Emphasis (SZLGE)

$$SZLGE\text{=}\frac{\sum_{i=1}^{N_{g}} \sum_{j=1}^{N_{s}} \frac{P\left(i,j\right)}{i^{2}j^{2}}}{N_{z}}$$

1. Small Zone High Gray-Level Emphasis (SZHGE)

$$SZHGE=\frac{\sum_{i=1}^{N_{g}} \sum_{j=1}^{N_{s}} \frac{P\left(i,j\right)i^{2}}{j^{2}}}{N_{z}}$$

1. Large Zone Low Gray-Level Emphasis (LZLGE)

$$LZLGE=\frac{\sum_{i=1}^{N_{g}} \sum_{j=1}^{N_{s}} \frac{P\left(i,j\right)j^{2}}{i^{2}}}{N_{z}}$$

1. Large Zone High Gray-Level Emphasis (LZHGE)

$$LZHGE=\frac{\sum_{i=1}^{N_{g}} \sum_{j=1}^{N_{s}} P\left(i,j\right)i^{2}j^{2}}{N_{z}}$$

1. Gray-Level Variance (GLV)

$$\begin{matrix} GLV=\sum_{i=1}^{N_{g}} \sum_{j=1}^{N_{s}} p\left(i,j\right)\left(i-\sum_{i=1}^{N_{g}} \sum_{j=1}^{N_{s}} p\left(i,j\right)i\right)^{2} \\ \end{matrix}$$

1. Zone-Size Variance (ZSV)

$$\begin{matrix} ZSV=\sum_{i=1}^{N_{g}} \sum_{j=1}^{N_{s}} p\left(i,j\right)\left(j-\sum_{i=1}^{N_{g}} \sum_{j=1}^{N_{s}} p\left(i,j\right)j\right)^{2} \\ \end{matrix}$$

- **Neighborhood gray-tone difference matrix (NGTDM):**

NGTDMs were calculated for the entire 3D volume, quantifying the difference between a gray value and the average gray value of its neighbors with Chebyshev distance δ [8]. Let: $n_{i}$ be the number of voxels in $X_{gl}$ with gray level $i$, $p_{i}$ be the gray level probability, $N_{g}$ be the number of discreet gray levels, $N_{p}$ be the total number of voxels in the ROI, $N_{g,p}$ be the number of gray levels where $p_{i}\neq0$, $N_{v,p}$ be the total number of voxels in $X_{gl}$, $A_{i}$ be the average gray level of the 26-connected neighbors around a center voxel with gray level $i$.

$$s_{i}=\left\{\begin{matrix} \sum_{i\in\left\{N_{i}\right\}} \left|i-\overline{A_{i}}\right|\text{ }if\text{ }n_{i}\neq0, \\ 0\text{ }otherwise \end{matrix}\right.$$

$$p_{i}=n_{i}/N_{v}$$

1. Coarseness

$$coarseness=\frac{1}{\sum_{i=1}^{N_{g}} p_{i}s_{i}}$$

1. Contra

$$contrast=\left(\frac{1}{N_{g,p}\left(N_{g,p}-1\right)}\sum_{i=1}^{N_{g}} \sum_{j=1}^{N_{g}} p_{i}p_{j}\left(i-j\right)^{2}\right)\left(\frac{1}{N_{v,p}}\sum_{i=1}^{N_{g}} s_{i}\right),where\text{ }p_{i}\neq0,p_{j}\neq0$$

1. Busyness

$$busyness=\frac{\sum_{i=1}^{N_{g}} p_{i}s_{i}}{\sum_{i=1}^{N_{g}} \sum_{j=1}^{N_{g}} \left|ip_{i}-jp_{j}\right|},where\text{ }p_{i}\neq0,p_{j}\neq0$$

1. Complexity

$$complexity=\frac{1}{N_{v,p}}\sum_{i=1}^{N_{g}} \sum_{j=1}^{N_{g}} \left|i-j\right|\frac{p_{i}s_{i}+p_{j}s_{j}}{p_{i}+p_{j}},where\text{ }p_{i}\neq0,p_{j}\neq0$$

1. Strength

$$strength=\frac{\sum_{i=1}^{N_{g}} \sum_{j=1}^{N_{g}} \left(p_{i}+p_{j}\right)\left(i-j\right)^{2}}{\sum_{i=1}^{N_{g}} s_{i}},where\text{ }p_{i}\neq0,p_{j}\neq0$$

# Supplementary note 3: 0.632+ bootstrap feature selection

The observed imaging data can be denoted as the matrix$\mathbf{X}=\left\{x_{i}:i=1\text{, 2,..., N}\right\}$. Also, a bootstrap sample is denoted as$\mathbf{X}^{*}=\left\{x_{i}^{*}:i=\text{1, 2,..., N}\right\}$, in which the input variables$x_{i}^{*}$is randomly drawn with the replacement from the available sample X. The generation of a large number B of randomly drawn bootstrap samples X*^b^ for b = 1, 2, …, B is used to estimate a statistical quantity of interest on the unknown true population distribution. The set of original data that do not appear in X*^b^ is denoted as X*^b^ (0). Prediction performance for the relevant features was evaluated by the 0.632+ bootstrap AUC is as follows:

**Table S1** Radiomics features type and number. Three extraction parameters with 48 parameter settings were conducted, thus the number of corresponding features is multiplied by 48 to bring forth a final number of 2068 in total.

| **Feature type** | **Count** | **Multipl.** | **Total** |
| --- | --- | --- | --- |
| Volume | 1 | - | 1 |
| Size | 1 | - | 1 |
| Solidity | 1 | - | 1 |
| Eccentricity | 1 | - | 1 |
| Global | 3 | $\times48$ | 144 |
| Gray-level co-occurrence matrix | 9 | $\times48$ | 432 |
| Gray-level run-length matrix | 13 | $\times48$ | 624 |
| Gray-level size zone matrix | 13 | $\times48$ | 624 |
| Neighborhood gray-tone difference matrix | 5 | $\times48$ | 240 |
|  |  |  | ***2068*** |

**Table S2** Prediction results of radiomics models from diverse VOIs on the training cohorts of two disease groups

| Dataset | Image | Radiomics model | Feature number | AUC | 95% CI | SEN | SPE | ACCU |
| --- | --- | --- | --- | --- | --- | --- | --- | --- |
| NPC group | CET1-w | Baseline | 15 | 0.813 | 0.794–0.832 | 74.8% | 78.4% | 76.9% |
|  |  | Erosion | 9 | 0.773 | 0.751–0.795 | 75.8% | 72.2% | 74.6% |
|  |  | Smoothing | 11 | 0.755 | 0.729–0.781 | 71.6% | 73.0% | 72.7% |
|  |  | Dilation | 10 | 0.769 | 0.746–0.792 | 74.8% | 75.0% | 75.4% |
|  |  | Dilation5 | 20 | 0.776 | 0.753–0.799 | 79.2% | 74.7% | 77.5% |
|  |  | Dilation7 | 14 | 0.808 | 0.786–0.830 | 77.8% | 75.6% | 77.2% |
|  | T2-w | Baseline | 11 | 0.733 | 0.707–0.759 | 73.8% | 72.4% | 73.8% |
|  |  | Erosion | 19 | 0.744 | 0.721–0.767 | 70.1% | 75.5% | 73.2% |
|  |  | Smoothing | 15 | 0.761 | 0.738–0.784 | 72.7% | 77.0% | 75.1% |
|  |  | Dilation | 20 | 0.768 | 0.747–0.789 | 74.3% | 75.5% | 75.3% |
|  |  | Dilation5 | 14 | 0.725 | 0.699–0.751 | 70.7% | 67.0% | 69.4% |
|  |  | Dilation7 | 18 | 0.793 | 0.770–0.816 | 78.2% | 74.1% | 76.8% |
| SLN group | DWI | Baseline | 15 | 0.821 | 0.791–0.851 | 81.2% | 77.4% | 79.6% |
|  |  | Erosion | 10 | 0.832 | 0.806–0.858 | 79.3% | 79.2% | 79.7% |
|  |  | Smoothing | 20 | 0.791 | 0.760–0.822 | 78.5% | 77.0% | 78.4% |
|  |  | Dilation | 20 | 0.826 | 0.800–0.852 | 74.3% | 78.9% | 77.8% |
|  |  | Dilation5 | 20 | 0.787 | 0.757–0.817 | 67.3% | 80.9% | 76.9% |
|  |  | Dilation7 | 8 | 0.839 | 0.813–0.865 | 75.5% | 75.1% | 76.0% |
|  | T2-FS | Baseline | 19 | 0.777 | 0.747–0.807 | 75.1% | 76.7% | 76.9% |
|  |  | Erosion | 20 | 0.762 | 0.730–0.794 | 75.2% | 76.3% | 76.7% |
|  |  | Smoothing | 4 | 0.844 | 0.819–0.869 | 75.1% | 79.1% | 78.2% |
|  |  | Dilation | 20 | 0.815 | 0.787–0.843 | 75.9% | 78.2% | 78.1% |
|  |  | Dilation5 | 18 | 0.827 | 0.801–0.853 | 78.3% | 71.3% | 75.2% |
|  |  | Dilation7 | 13 | 0.843 | 0.816–0.870 | 83.1% | 75.6% | 79.4% |

*Erosion* erosion operation, *Smoothing* smoothing operation, *Dilation* dilation with structural element radius size of 3, *Dilation5* dilation with structural element radius size of 5, *Dilation7* dilation with structural element radius size of 7, *AUC* area under receiver operating characteristic curve, *CI* confidence interval, S*EN* sensitivity, *SPE* specificity, *ACCU* accuracy

**Table S3** Radiomics features for the Dilation7 (dilation with structural element radius size of 7) model as well as the ICCs for metastasis estimation in nasopharyngeal carcinoma. ICC1 is calculated from Dilation7 on VOIs while ICC2 represents the average of ICCs from all operations on VOIs.

| Parameter settings | | | | ICC | |
| --- | --- | --- | --- | --- | --- |
| *Scale* | *Quant. alg* | *Ng* | Feature name | ICC1 | ICC2 |
| 5 | Equal | 64 | GLCM_Dissimilarity | 0.07 | 0.43 |
| 3 | Lloyd | 16 | NGTDM_Coarseness | 0.61 | 0.80 |
| 4 | Lloyd | 32 | GLSZM_SZHGE | 0.29 | 0.36 |
| pixelW | Equal | 64 | GLSZM_SZE | 0.62 | 0.85 |
| pixelW | Equal | 16 | GLSZM_GLV | 0.37 | 0.65 |
| pixelW | Equal | 8 | GLSZM_ZSV | 0.72 | 0.86 |
| pixelW | Equal | 64 | GLSZM_LZLGE | 0.08 | 0.50 |
| 5 | Equal | 8 | Global_Skewness | 0.33 | 0.44 |
| 4 | Lloyd | 16 | GLSZM_ZSV | 0.29 | 0.43 |
| pixelW | Equal | 16 | GLSZM_ZP | 0.58 | 0.82 |
| 1 | Equal | 16 | GLSZM_HGZE | 0.39 | 0.68 |
| 5 | Equal | 64 | GLCM_Contrast | 0.07 | 0.44 |
| pixelW | Lloyd | 32 | GLSZM_SZE | 0.79 | 0.83 |
| 1 | Lloyd | 8 | GLSZM_SZE | 0.36 | 0.40 |
| pixelW | Equal | 32 | GLRLM_GLV | 0.59 | 0.72 |

*Scale* isotropic voxel size, *Quant. Alg* quantization algorithms, *Ng* quantization of gray levels, *pixelW* initial in-plane resolution

**Reference**

1. Vallières M, Freeman CR, Skamene SR, El NI. A radiomics model from joint FDG-PET and MRI texture features for the prediction of lung metastases in soft-tissue sarcomas of the extremities. Phys Med Biol. 2015;60(14):5471-5496. https://doi.org/10.1088/0031-9155/60/14/5471.

1. Li Q, Bai H, Chen Y, Sun Q, Liu L, Zhou S, et al. A Fully-Automatic Multiparametric Radiomics Model: Towards Reproducible and Prognostic Imaging Signature for Prediction of Overall Survival in Glioblastoma Multiforme. Sci. Rep. 2017;7(1):14331. <https://doi.org/10.1038/s41598-017-14753-7.>

3. Haralick R M. Texture features for image classification. IEEE Transactions on Systems, Man, and Cybernetics. 1973;SMC-3(6):610-621. <https://doi.org/10.1109/TSMC.1973.4309314.>

4. Galloway MM. Texture analysis using gray level run lengths. Computer Graphics & Image Processing. 1975;4(2):172-179. https://doi.org/10.1016/S0146-664X(75)80008-6.

5. Chu A, Sehgal C M, Greenleaf J F. Use of gray value distribution of run lengths for texture analysis. Pattern Recog. Lett. 1990;11(6):415-419. <https://doi.org/10.1016/0167-8655(90)90112-F.>

6. Dasarathy B V, Holder E B. Image characterizations based on joint gray level—run length distributions. Pattern Recog. Lett. 1991;12(8):497-502. https://doi.org/10.1016/0167-8655(91)80014-2.

7. Thibault G, Fertil B, Navarro C, Pereira S, Cau P, Levy N, et al. SHAPE AND TEXTURE INDEXES APPLICATION TO CELL NUCLEI CLASSIFICATION. Int J Pattern Recogn. 2013;27(1):1357002. https://doi.org/10.1142/S0218001413570024.

8. Amadasun M, King R. Textural features corresponding to textural properties. IEEE Transactions on Systems, Man and Cybernetics. 1989;19(5):1264-1274. https://doi.org/10.1109/21.44046.
